# Modern applications for a total sulfur reduction distillation method - what’s old is new again

**DOI:** 10.1186/1467-4866-15-4

**Published:** 2014-04-22

**Authors:** Gail L Arnold, Benjamin Brunner, Inigo A Müller, Hans Røy

**Affiliations:** 1 Max Planck Institute for Marine Microbiology, 28359 Bremen, Germany; 2 Center for Geomicrobiology, Department of Bioscience, Aarhus University, 8000 Aarhus, Denmark; 3 Department of Geological Sciences, University of Texas at El Paso, El Paso, TX, USA

## Abstract

**Background:**

The use of a boiling mixture of hydriodic acid, hypophosphorous acid, and hydrochloric acid to reduce any variety of sulfur compounds has been in use in various applications since the first appearance of this method in the literature in the 1920’s. In the realm of sulfur geochemistry, this method remains a useful, but under-utilized technique. Presented here is a detailed description of the distillation set-up and procedure, as well as an overview of potential applications of this method for marine sulfur biogeochemistry/isotope studies. The presented applications include the sulfur isotope analysis of extremely low amounts of sulfate from saline water, the conversion of radiolabeled sulfate into sulfide, the extraction of refractory sulfur from marine sediments, and the use of this method to assess sulfur cycling in Aarhus Bay sediments.

**Results:**

The STrongly Reducing hydrIodic/hypoPhosphorous/hydrochloric acid (STRIP) reagent is capable of rapidly reducing a wide range of sulfur compounds, including the most oxidized form, sulfate, to hydrogen sulfide. Conversion of as little as approximately 5 micromole sulfate is possible, with a sulfur isotope composition reproducibility of 0.3 permil.

**Conclusions:**

Although developed many decades ago, this distillation method remains relevant for many modern applications. The STRIP distillation quickly and quantitatively converts sulfur compounds to hydrogen sulfide which can be readily collected in a silver nitrate trap for further use. An application of this method to a study of sulfur cycling in Aarhus Bay demonstrates that we account for all of the sulfur compounds in pore-water, effectively closing the mass balance of sulfur cycling.

## Background

In recent years, it has become evident that the biogeochemistry of sulfur is far more complex than previously thought. There are ample discoveries of so far unrecognized sulfur transformations, such as i) sulfate generation in methanic sediments well below the main sulfate zone, the so-called “cryptic sulfur cycle” [1], ii) potential reduction of sulfate to zero-valent sulfur by methanotrophic archaea that utilize biochemical pathways different from classical bacterial dissimilatory sulfate reduction [2] and iii) the oxidation of reduced sulfur by cable forming bacteria [3], or iv) sulfur cycling in the oceanic crust [4]. These findings highlight three needs: First, the highly sensitive tracing of sulfur transformations with the help of radioactive ^35^S-labeled compounds is more essential than ever; second, progress in our understanding of sulfur cycling without closed sulfur and sulfur isotope mass balances can hardly be achieved because potentially pivotal sulfur pools and fluxes escape our detection, and third, the sulfur isotope composition of sulfur compounds at very low concentrations must become accessible to allow for meaningful interpretations of so far hidden sulfur transformations. With the **ST**rongly **R**educing hydr**I**odic - hypo**P**hosphorous – hydrochloric acid (STRIP) method (presented here, modified from [5-8]), these needs can be covered to a substantial degree. In the following, it will be demonstrated how the sulfur isotope composition of sulfate at extremely low concentration from highly saline environments can be determined by conventional gas-source isotope ratio mass spectrometry with the help of the STRIP method; it will be shown how the STRIP method can be used for the production of ^35^S-labeled sulfide from the commonly commercially available ^35^S-sulfate tracer; and examples will be given on how this method can be used to obtain closed sulfur isotope mass balances, as well as for the assessment of undetected sulfur pools.

The use of hydriodic acid (HI) to reduce sulfur compounds can be found in the literature as far back the 1920’s [9] continuing to more recent applications, e.g. Shan and Chen [10]. However, HI alone will not liberate all sulfur species, leaving pyrite and organically-bound sulfur behind [10,11]. The combination of HI with hypophosphorous acid (H_3_PO_2_) and hydrochloric acid (HCl) to convert sulfur species including sulfate to H_2_S for analysis was first introduced by Luke [5] who used this method to determine the sulfur content of rubber. Many subsequent variations in the following decades adjusted both the initial concentrations and relative proportions of reagents to find an optimal combination that would yield good results while minimizing the amount of the relatively expensive HI used (Table 1; [5,11]).

**Table 1:** Commonly referenced literature for STRIP reagent in sulfur geochemistry and reagent details

	**Luke, 1943**	**Pepkowitz & Shirley 1951**	**Thode et al. 1961**	**Forrest & Newman 1977**	**Johnston et al. 2007**	**Arnold et al. This study**
Hydriodic acid	160 ml	100 ml	500 ml	500 ml	125 ml	200 ml
	s.g. = 1.7	47%	s.g. = 1.7	48%	n.o.s.	s.g. =1.7, 57%
	45 ml	40 ml	245 ml	245 ml	61 ml	100 ml
Hypophosphorous acid	n.o.s.	H_3_PO_2_	H_3_PO_2_	H_3_PO_3_^*^	H_2_PO_4_^*^	H_3_PO_2_
	50%	30%	50%	50%	n.o.s.	50%
Hydrochloric acid	160 ml	160 ml	816 ml	816 ml	205 ml	330 ml
	n.o.s.	conc	conc	conc	n.o.s.	36%

*All references call for the use “hypophosphorus” acid, variations in chemical formula are likely typographical errors. n.o.s. = not otherwise specified, s.g. = specific gravity.

In the realm of sulfur isotope geochemistry, the most commonly referenced work using this method is from Thode et al. [7] who used the STRIP distillation to convert barium sulfate (BaSO_4_) to silver sulfide (Ag_2_S) for subsequent isotopic analysis. Simultaneously a method that is similar in its applications but employs a mixture of stannous chloride with phosphoric acid (the Kiba reagent) was developed [12] and subsequently refined [13]. The later advent of the direct conversion of BaSO_4_ to SO_2_ for sulfur isotope analysis removed the necessity to covert the BaSO_4_ to Ag_2_S for most applications [14]. Besides its application in the conversion of sulfur bearing samples to Ag_2_S for subsequent fluorination techniques for multiple sulfur isotope analysis (e.g. [15]) the STRIP method has fallen into dis-use. There is a suite of reasons why this should not be the case, examples of which will be presented in this study. The aim of this contribution is not a comprehensive test of the STRIP method as recent publications have been with regards to the chromium (Cr)-distillation [16,17] but means to bring this highly useful technique with a complete method description back into view in light of its many potential applications in the realm of sulfur isotope biogeochemistry.

## Methods

### Preparation of the reducing reagent

Preparations of and distillations using the STRIP reagent should be done in a well-ventilated hood. The reducing reagent recipe here follows that of Thode et al. [7]. Note that the concentration reported for hydriodic acid has varied through the years, as well as the chemical formula for hypophosphorous acid reported in the literature (Table 1). With regard to the concentration of hydrochloric acid used in the preparation of the reagent, existing literature only specifies “concentrated” (Table 1; [5-8,15]). The purpose of the hydrochloric acid has been suggested to increase acidity and volume, such that less hydriodic acid is required [11]. In the course of this study, 32%, 36% or 37% hydrochloric acid has been used at one time or another and no difference in the performance of the reagent has been observed.

**Figure 1:**
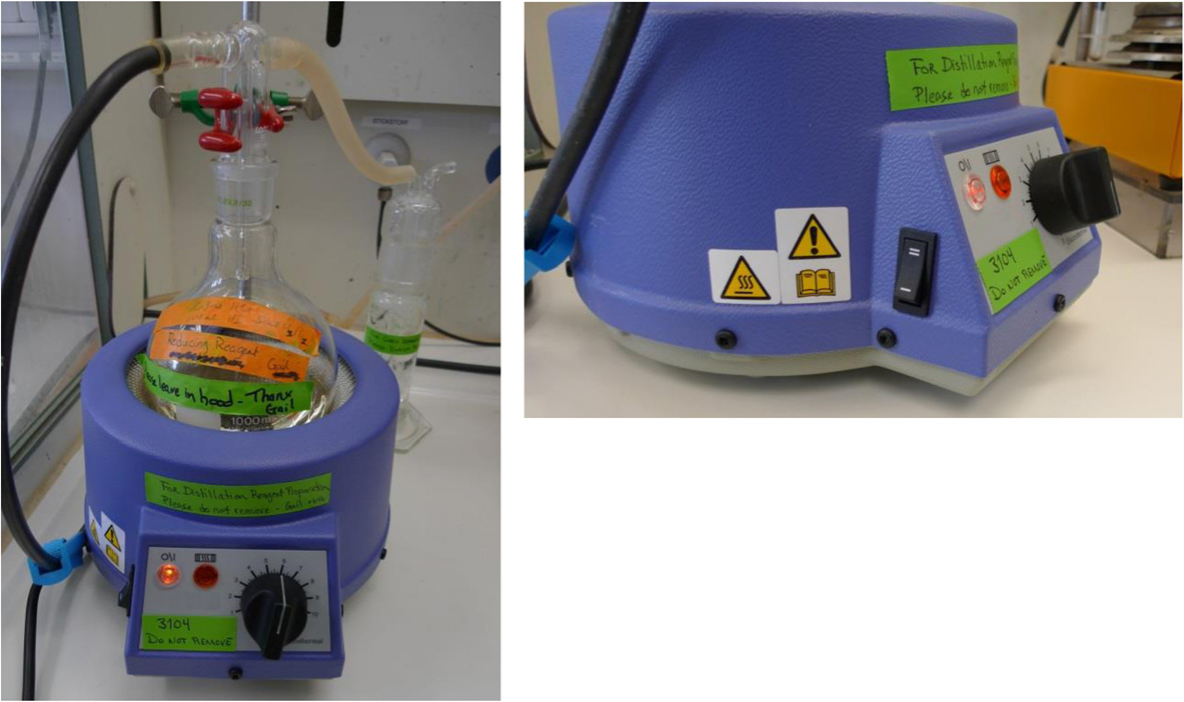
**STRIP reagent preparation.** The reagent preparation generates a lot of acid fumes, therefore the flask top must be firmly set and clamped in place and a gas wash flask attached to the outlet. With our set-up, we set the switch on the side to the “II” position and the dial to the 6 setting. The unit took approximately 30 minutes to bring the acid to a boil.

The reagent should be prepared under a stream of nitrogen (N_2_) or other inert gas to avoid oxidation with O_2_. The reagent preparation will generate a lot of acid fumes, thus the flask top must be firmly set and clamped in place (or it will pop up during the boiling and spit hot acid around) and a water trap, ideally a gas wash bottle, should be connected to the outlet of the flask (Figure 1). In a 1000 ml round bottom flask, 200 ml HI (57%), 100 ml H_3_PO_2_ (50%), and 330 ml HCl (concentrated) are combined, and a small spoonful of boiling stones is added to the flask. The boiling stones should be silicon carbide [5,18] or some other suitably dense material. PTFE boiling chips are unsuitable as they will float on top of the acid mixture. The flask is placed on a heating mantle, the gas lines assembled, and the N_2_ gas set to as gentle a stream as possible. The mixture is brought to a vigorous boil and boiled for 60 minutes to remove any sulfur contamination present in the reagents. The mixture should be allowed to cool under the N_2_ stream. Once the mixture is cool, it should be stored in a brown glass bottle to avoid photo-oxidation of HI [5].

As the STRIP reagent may oxidize over time, note the date of the preparation on the bottle and use the reagent within 6 months of its preparation. Smaller batches can be made. Due to the vigorous nature of the boiling, reagent batches should not exceed a total volume of 630 ml per 1000 ml flask.

### Preparation of test samples

All samples, solid or aqueous, processed during the development of this method started from the same commercially available sodium sulfate (Na_2_SO_4_, Sigma-Aldrich Lot #S23924-206). A sulfate control solution (~25 mM) was made by dissolving a known amount of Na_2_SO_4_ in ultraclean water. Method development samples are numbered sequentially and labeled either with a “C” (for control) or “S” (for standard) prefix. The C series were run solely for method development. The S series were run as internal standards during the Aarhus Bay study.

### Distillation apparatus

**Figure 2:**
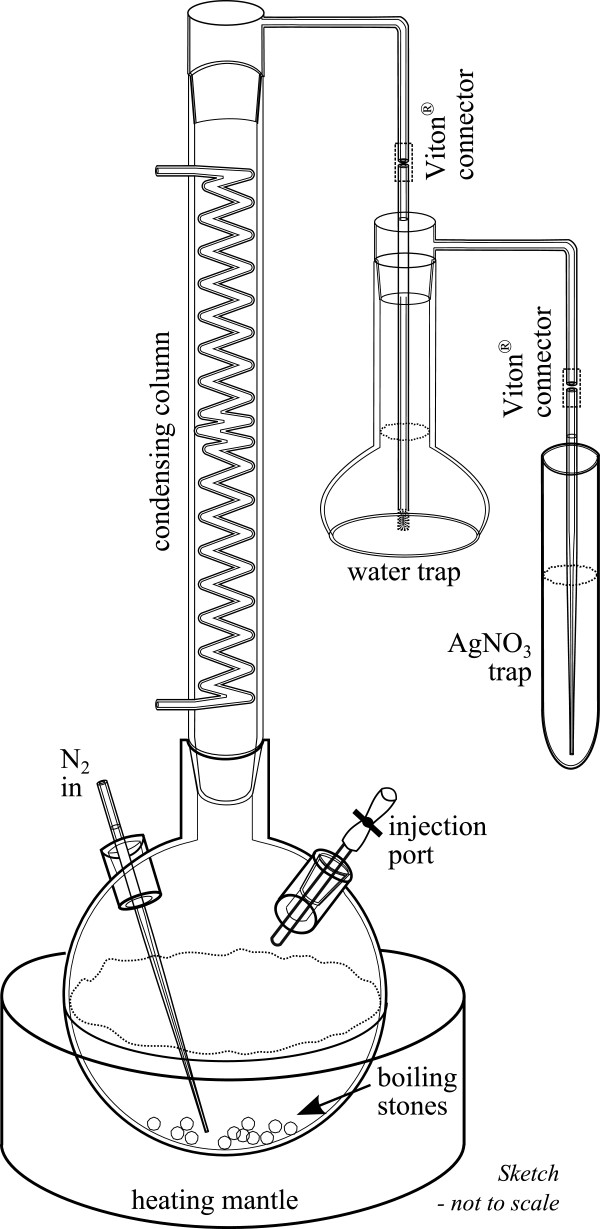
**Distillation apparatus.** Connections are either ground glass surface or connected with short segments of Viton® tubing. First trap = 45 ml water. Second trap = 10 ml AgNO_3_. Fluid levels indicated by dotted line.

Samples are distilled in a 100 ml round bottom, 3-neck flask, connected to a condensing reflux column with recirculating chilled (4°C) water (Figure 2). The outlet of the condensing column is followed by a water trap, which is then followed by the trapping solution. It is advised to keep the amount of tubing between individual glass parts as minimal as possible to limit exposure of tubing to the reagent fumes. First applications of the STRIP distillation were the reduction of sulfur in rubber samples [5], so this mixture may be detrimental to any tubing it comes in contact with. Silicon tubing, which in this study was originally used to connect the outlet of the 1000 ml flask to the gas wash bottle during the preparation of the reagent, was rapidly degraded after a few uses. Viton® tubing was used for the connections in the distillation apparatus and has shown the most durability.

### Distillation method

This reagent will reduce all sulfur species (sulfate, sulfide, elemental S, polysulfides, all sulfur intermediate species, and any other sulfur compounds present) of a sample to H_2_S gas. To begin sample preparation, a sample (aqueous or solid) is transferred into the distillation flask and dried. Aqueous samples that contain sulfide should be fixed with zinc acetate (ZnAc) prior to the drying procedure to avoid degassing of sulfide and/or oxidation of sulfide. The use of ZnAc as the precipitation agent instead of zinc chloride has the advantage that the protons liberated from bisulfide and hydrogen sulfide can form a relatively weak acid (acetic acid) which degasses upon drying. If only chloride is present, the drying procedure could result in a more acidic sample (hydrochloric acid), which – unlike acetic acid – has the potential to liberate sulfide from zinc sulfide. Larger volume aqueous samples or wet sediments can be dried overnight in a 70°C oven. Smaller volume aqueous samples may be dried in the distillation flask with gentle heating from the hot plate. The sample must be dried completely such that no water remains. Any water from the sample will condense in the column and drip back into the reagent where it immediately vaporizes due to flash boiling. This and the subsequent rapid condensation of water vapor in the cooling column creates large and dangerous pressure “bumps” and back fluxing of vapors and trap fluids. After drying of the samples, a few boiling stones (see section on preparation of STRIP reagent) are added to the sample flask and the distillation apparatus is assembled, cooling water and N_2_ gas flow started. Because the acid mixture is brought rapidly to a boil, it is important to make sure that the cooling water has reached a stable temperature prior to heating the sample. Approximately 20 to 30 ml of STRIP reagent are added to the sample via an inlet port on one side of the distillation flask (Figure 2). The reagent-sample mixture is then brought to a boil as swiftly as possible. Distillation at low temperatures will result in lower yields [5]. Once the sample-reagent mixture is boiling any sulfur present is reduced to H_2_S. The evolved H_2_S and acid fumes are transported by the N_2_ gas stream first into the water trap which serves as a trap for acid fumes and subsequently into a 10 ml 1 M AgNO_3_ trap where the H_2_S is trapped and converted to Ag_2_S. Despite the presence of a water trap, a certain amount of acid fumes (likely HI) will make it to the silver nitrate (AgNO_3_) trap, increasing the apparent Ag_2_S yield. The excess precipitate is grey/black in color and is not dissolved with NH_4_OH suggesting that the excess is not AgCl, but may be something else, for instance AgI or a phosphorus compound. Samples are distilled for 1 hour, after which the hot plate is turned off and the N_2_ flow in maintained for another hour to 1) allow the very hot boiling acid to cool sufficiently to handle safely and 2) to ensure that all H_2_S in the system has been carried to the final trap. Once cool, the AgNO_3_ trap is removed and the Ag_2_S is separated and thoroughly washed with ultra-clean water and dried. We recommend turning off the N_2_ flow only after the traps have been removed from the distillation assembly. This ensures that there is no back-aspiration of fluids resulting in loss of sample.

### Quantification of distilled sulfur and isotope analysis

The STRIP distillation samples always have greater apparent Ag_2_S yields than is predicted from the amount of sulfur distilled. For the determination of the sulfur isotope compositions of pure substances, i.e., Na_2_SO_4_, BaSO_4_, or Ag_2_S standards, 0.2–0.4 mg are weighed into tin capsules. Approximately 0.5 mg of vanadium pentoxide (V_2_O_5_) is added to both standards and samples. Greater amounts are weighed in for the STRIP distillation samples. First, the weight of the entire ‘Ag_2_S’ precipitate is recorded and then an appropriate aliquot of sample is weighed into the tin capsule. For example, with samples of known sulfur content such as pore-waters where the sulfate and sulfide concentrations have been determined prior to the preparation of the isotope analysis samples, if the predicted mass of Ag_2_S is 1.0 mg but 3.0 mg of precipitate are recovered, we then know that the Ag_2_S in the sample is diluted by a factor of 3, thus 3 times more sample needs to be weighed in (0.9 mg instead of 0.3 mg). This technique has its limitations as the amount of sample that can fit in a tin cup is limited. Up to 2.0 mg of precipitate has been run as a single sample with no detectable ill-effects such as poor combustion, double peaks or extended peak tailing during isotopic analysis. For samples where the sulfur content is not known at the time of isotopic analysis, a second single-step Cr-distillation can be used to purify the STRIP method precipitate [17,19], yielding only Ag_2_S, which can then be weighed in as appropriate. The samples and standards were combusted with an elemental analyzer (EURO EA Elemental Analyzer, set to 1060°C) to produce SO_2_. A helium stream carried the evolved SO_2_ through a GC column and Finnigan Conflo III into a Finnigan Delta V stable isotope ratio mass spectrometer (IRMS). The sulfur isotope measurements were calibrated with reference materials NBS 127 (δ^34^S = +20.3‰) and IAEA-SO-6 (δ^34^S = -34.1‰). The standard error (1σ) for replicate measurements of a laboratory standard was less than 0.2‰. Quantification of sulfur content is obtained by comparing the integrated peaks areas (in V∙s, detected and quantified by IRMS software) samples against the peak areas produced from known amounts of standards, followed by the calculation of the total amount of distilled sulfur from the comparison of the weight of the combusted sample to the total weight of the precipitate.

## Results and discussion

### Choice of trapping solution

The most recent studies using the STRIP distillation method describe the conversion of BaSO_4_ to Ag_2_S as a process where ZnAc is used in place of AgNO_3_ as the sulfide trap. In a subsequent separate step, the produced ZnS is then converted to Ag_2_S which can be used for sulfur isotope analysis via fluorination [15,20,21]. The utilization of ZnAc as the initial trapping solution is problematic as the acid fumes from the distillation that are not condensed in the cooling column rapidly acidify all subsequent solutions in the distillation including the final trap. If the ZnAc trap is exposed to acidification, then any ZnS that had been precipitated will be re-dissolved and sulfur lost as H_2_S. The use of ZnAc as the trapping solution was initially tested as part of the development of this study, but sulfur isotope composition results were offset from the known composition and the reproducibility was less than desirable (Table 2). For these reason we moved to the use of AgNO_3_ as the trapping solution.

**Table 2:** Results of STRIP distillation with 10 ml 5% ZnAc trap

**identifier**	**Amount (mg)**	**δ**^**34**^**S**_**VCDT**_**‰**
Na_2_SO_4_ (solid, distilled)	0.212	-2.3
Na_2_SO_4_ (solid, distilled)	0.392	-2.5
Na_2_SO_4_ (solid, distilled)	0.406	-2.1
Na_2_SO_4_ (solid, distilled)	0.230	-2.4
	**Average**	**-2.3**
	**stdev (1SD)**	**0.2**
C1	0.224	-1.7
C2	0.295	-1.9
C3	0.745	-1.1
C4	0.650	-1.8
C5	0.269	-2.8
C6	0.670	-2.2
C7	0.370	-2.1
C8	0.240	-1.7
C9	0.303	Sample lost
C10	0.274	-2.2
C11	0.228	-2.3
C12	0.315	-1.9
C13	0.351	-1.7
	**Average**	**-2.0**
	**stdev (1SD)**	**0.4**

C1 – C13 samples are all from a STRIP distillation of 1.0 ml of ~25 mM sulfate solution.

### Reproducibility of the STRIP distillation method using a AgNO_3_ trap

Poor reproducibility was observed when the AgNO_3_ trap volume was changed from 10 to 5 ml. Stoichiometry is not the only consideration when designing the trap volume and concentration. The length and speed at which the H_2_S travels with the carrier gas N_2_ through the trap needs be taken into consideration as well. Excessive carrier gas flow or too short a path will result in poorer data reproducibility (Table 3).

**Table 3:** **Results from 5 vs 10 ml AgNO**_**3 **_**trap**

**5 ml 0.1 M AgNO**_**3**_				**10 ml 0.1 M AgNO**_**3**_			
**Identifier**	**Amount (mg)**	**Area (V∙s)**	**δ**^**34**^**S**_**VCDT**_**‰**	**Identifier**	**Amount (mg)**	**Area (V∙s)**	**δ**^**34**^**S**_**VCDT**_**‰**
S5	0.385	17.5	-1.6	C20	1.943	27.6	-2.6
S6	0.399	17.3	-2.0	C21	1.533	27.4	-2.4
S7	0.350	7.1	-1.2	C24	1.520	35.6	-2.0
S8	0.383	14.0	-1.7	C25	1.630	37.4	-2.3
S9	0.476	20.6	-1.7	C32a	1.560	23.5	-1.7
S10	0.377	8.7	-0.5	C32b	1.595	22.7	-1.8
S11	0.353	6.3	-1.0	C33a	1.588	26.1	-2.1
S12	0.378	10.0	-0.8	C33b	1.582	19.0	-2.3
S13	0.423	12.4	-2.0	C36	2.502	43.3	-2.1
S14	0.451	17.1	-3.0	C37	2.562	48.4	-2.2
S15	0.944	27.5	-2.2	C40	2.388	44.3	-2.4
		**Average**	**-1.6**	C41	2.479	49.3	-2.5
		**stdev (1SD)**	**0.7**	C42	2.734	59.0	-2.4
						**Average**	**-2.2**
						**stdev (1SD)**	**0.3**

S & C series samples are all from STRIP distillation of 0.2 ml of ~25 mM sulfate solution (~ 5 μmol of total sulfur).

In order to test the yield and reproducibility of the STRIP distillation method with the appropriate 10 AgNO_3_ trap, sulfate in varying quantities and form was distilled. Results for the reproducibility and integrity of the STRIP distillation method are summarized in Table 4. Repeated distillations of 1 ml and 0.2 ml of the control solution, 25 and 5 μmole of sulfate-sulfur, respectively, yielded results of δ^34^S_VCDT_ = -2.5 ± 0.5‰ and -2.2 ± 0.3‰ (Table 4), respectively and -2.3 ± 0.3‰ collectively. Barium sulfate was directly precipitated from the control solution and directly analyzed, yielding δ^34^S_VCDT_ = -2.4 ± 0.1‰. The solid sodium sulfate was also analyzed directly, yielding a δ^34^S_VCDT_ = -2.9 ± 0.4‰. Peak areas for the Na_2_SO_4_ solid were 23% smaller than the average for either Ag_2_S or BaSO_4_ standards, indicating poor combustion in the reactor, likely contributing to the shifted sulfur isotope composition of the Na_2_SO_4_ relative to the BaSO_4_ precipitated from the solution and the Ag_2_S produced from the distillation of the control solution (Table 4). Regardless of the complications associated with the isotope analysis of solid Na_2_SO_4,_ there is no observed offset between the results obtained with the BaSO_4_ precipitation method and the STRIP distillation.

**Table 4:** **Summary STRIP distillation results for Na**_**2**_**SO**_**4**_

**Identifier**	**Amount (mg)**	**Area (V∙s)**	**δ**^**34**^**S**_**VCDT**_**‰**	**mmol S**	**Area/mmol**	
Na_2_SO_4_ (solid)	0.366	54.9	-2.4	0.0026	21313	
Na_2_SO_4_ (solid)	0.368	57.1	-2.9	0.0026	22037	
Na_2_SO_4_ (solid)	0.386	61.7	-2.8	0.0027	22709	
Na_2_SO_4_ (solid)	0.387	77.5	-3.0	0.0027	28454	
Na_2_SO_4_ (solid)	0.364	87.2	-2.1	0.0026	34028	
Na_2_SO_4_ (solid)	0.481	74.8	-3.2	0.0034	22088	
Na_2_SO_4_ (solid)	0.352	83.5	-2.6	0.0025	33691	
Na_2_SO_4_ (solid)	0.364	71.5	-3.3	0.0026	27903	
Na_2_SO_4_ (solid)	0.489	96.4	-3.1	0.0034	28001	
		**Average**	**-2.8 +/-0.4**		**26692**	
C-14 (Na_2_SO_4_, solid, distilled)	0.524	31.7	-2.3			
C-15 (Na_2_SO_4_, solid, distilled)	0.481	30.7	-2.6			
		**Average**	**-2.5 +/-0.2**			
BaSO_4_ (from control solution)	0.381	55.7	-2.4	0.0016	34152	
BaSO_4_ (from control solution)	0.381	55.6	-2.3	0.0016	34050	
BaSO_4_ (from control solution)	0.474	66.5	-2.6	0.0020	32764	
BaSO_4_ (from control solution)	0.384	54.2	-2.4	0.0016	32969	
		**Average**	**-2.4 +/-0.1**		**33484**	
	**Amount (mg)**	**Area (V∙s)**	**δ**^**34**^**S**_**VCDT**_**‰**	**mmol S IRMS**	**Fraction sample weighed in**	**Distillation yield**
**1 ml 25 mM sulfate**						
C18	0.431	25.0	-2.3	0.0007	-	-
C19	0.590	36.8	-2.9	0.0011	-	-
C22	0.429	40.1	-3.4	0.0012	-	-
C23	0.592	57.6	-2.0	0.0017	-	-
C34	0.472	33.7	-2.3	0.0010	0.04	96%
C35	0.606	47.3	-2.2	0.0014	0.06	103%
		**Average**	**-2.5 +/-0.5**			
**0.2 ml 25 mM sulfate**						
C20	1.943	27.6	-2.6	0.0008	-	-
C21	1.533	27.4	-2.4	0.0008	-	-
C24	1.520	35.6	-2.0	0.0011	-	-
C25	1.630	37.4	-2.3	0.0011	-	-
C32a	1.560	23.5	-1.7	0.0007	-	-
C32b	1.595	22.7	-1.8	0.0007	-	-
C33a	1.588	26.1	-2.1	0.0008	-	-
C33b	1.582	19.0	-2.3	0.0006	-	-
C36	2.502	43.3	-2.1	0.0013	0.26	100%
C37	2.562	48.4	-2.2	0.0014	0.29	98%
C40	2.388	44.3	-2.4	0.0013	0.35	77%
C41	2.479	49.3	-2.5	0.0015	0.30	100%
C42	2.734	59.0	-2.4	0.0018	0.29	121%
		**Average**	**-2.2 +/-0.3**			
C-38 (0.02 ml)	4.165	7.9	-2.0	0.0002	0.45	105%
C-39 (0.02 ml)	3.899	6.9	-0.9	0.0002	0.51	81%

-weights were not recorded/yields could not be calculated.

In summary, the use of AgNO_3_ as the trapping solution alleviated all problems associated with sample loss and lack of reproducibility. Although AgNO_3_ as a trapping agent does have the drawback of excess precipitate forming in the trap, the excess mass has not proved at all detrimental to further isotopic analyses and if necessary can be removed by a second distillation using the Cr-distillation [17,19] method.

### Caveats of the method

If nitrate is present, the H_2_S may be oxidized to elemental sulfur in the condensing column [6]. Care should also be taken with samples high in organic matter content as the presence of organic matter makes the distillation reaction more exergonic and earlier applications of this method contained steps to remove organic compounds prior to the addition of the STRIP reagent [5,6,11]. It remains unclear whether the initial oxidation of refractory organic compounds prior to the treatment of the sample with the STRIP distillation in early studies was to improve the sulfur yield during distillation or to avoid potentially dangerous distillation conditions.

### Examples for the application of the STRIP method

In the following, we present four different examples in which the STRIP method can be used, and highlight specific adjustments in the methodology that have to be made for obtaining an optimal outcome.

### Preparation of low sulfate concentration pore-water samples for sulfur isotope analysis

At concentrations below ~0.5 mM sulfate, the traditional method of precipitating BaSO_4_ from a sample of seawater, pore-water, or marine or fresh-water type media by the addition of barium chloride (BaCl_2_) frequently fails. While the use of ion selective resin is useful to pre-concentrate sulfate in fresh-water samples [22-24], the resin based method remains unfavorable in marine and marine-type media due to the high and close affinity of the resin for chloride and sulfate. Recently, the use of multiple collector – inductively coupled plasma mass spectrometry (MC-ICPMS) has been developed for the analysis of extremely small quantities of sulfate [25,26]. Although sulfur isotope analysis by MC-ICPMS lowers the detection limit for sulfur isotope analysis by several orders of magnitude (down to ~5 nmol S), the sample preparation prior to the MC-ICPMS analysis still requires resin based chemistry and there are less than a handful of facilities that specialize in this instrumentation and methodology [25,27]. The STRIP reagent presented here offers a reliable, straightforward, affordable and widely accessible alternative, which can be even employed when the BaCl_2_ method or resin pre-concentration method is not useful. As the STRIP reduction-distillation method will reduce all sulfur present in the sample, if other sulfur species (e.g. sulfide) are present, care must be taken to separate the sulfur species prior to the beginning of the distillation. Sulfide that may be present in the sample can be separated from the sulfate through the addition of ZnAc. Directly after sample collection 0.2 ml of 20% ZnAc per 1.0 ml sample is added. Sulfide present in the sample will precipitate as solid zinc sulfide (ZnS). The ZnS and elemental sulfur is separated from the supernatant by vacuum filtration (0.2 μm). The supernatant, which at this point should contain mostly sulfate, but may also contain sulfur intermediates such as thiosulfate or sulfite, can then be distilled to Ag_2_S for further analysis using the STRIP distillation method. If the presence of significant quantities of thiosulfate or sulfite is suspected, these compounds can be removed via acidification. Thiosulfate will disproportionate into sulfur dioxide and elemental sulfur and sulfite will be converted to sulfur dioxide. The elemental sulfur can then be filtered out and the sulfur dioxide can be degassed from the sample.

### Conversion of ^35^S-sulfate to ^35^S-sulfide tracer

Sulfide is a substrate for many oxidative S-cycling processes and an important reactant for the synthesis of other ^35^S-labeled compounds. The reductive distillation of ^35^S-sulfate to ^35^S-sulfide tracer is principally the same as for any other aqueous sulfur distillation, given proper safety measures required for the handling of radioactive materials are taken. An appropriate amount of ^35^S-sulfate tracer is transferred to the distillation flask, gently dried and distilled. Because the sulfate concentration in the radio-tracer is so low, the STRIP distillation should be performed using a 10 ml or larger de-oxygenated AgNO_3_ sulfide trap to ensure that 100% of the converted tracer is trapped. The entire contents of the trap can then be dried into a new distillation flask for a subsequent Cr-distillation [17,19]. The adjustment here is that the trapping solution for the Cr-distillation should be changed, because is it preferable to obtain a dissolved ^35^S sulfide tracer instead of a solid Ag_2_ ^35^S product. Hence, sodium hydroxide (NaOH, also de-oxygenated) should be used as the trapping solution. There are two considerations to keep in mind when calculating the volume and strength of the NaOH solution to be used. First, and most straight forward, is the stoichiometric balance needed to trap and convert the evolved H_2_S to Na_2_S in solution. Second, the volume of the trap must be optimized such that the desired activity per volume is achieved. An earlier study directly used 1 M NaOH as the trapping solution with a STRIP distillation method and reported satisfactory tracer recovery (~100%, [28]). Our attempts at replicating this yielded results in the 30 to 50% recovery range. As such, we suggest the use of AgNO_3_ as the STRIP distillation trap, followed by subsequent conversion to a dissolved tracer form using a Cr-distillation and appropriate trap.

### Solid phase samples – sediment and rock

The analysis of the quantity and sulfur isotope composition of acid-volatile sulfur and chromium-reducible sulfur (CRS) in sediments and rocks ( [17] and references therein) is common to many sulfur geochemistry studies. The STRIP distillation can be used for the determination of the sulfur content and isotope composition of bulk sediment and rock samples, or can be used as a final step in a sequential extraction scheme, for example in series with a Cr-distillation step, to determine the refractory/residual sulfur phases. For both rock and sediment, the sample should be well homogenized and ground to a fine powder. Sediment and rock samples high in carbonate content should be de-carbonated prior to distillation. Wet sediment needs to be dried prior to distillation.

We applied the STRIP reagent to marine sediments in a sequential extraction. After thawing, the samples (~5 g) were covered with 50–100 ml of 2 M sodium chloride solution. Over at least 24 hours, the sediment was repeatedly brought in suspension by stirring. Subsequently, the supernatant was decanted and the sample was washed with deionized water by three consecutive centrifugation-resuspension steps. The sodium chloride leaching step serves to remove sulfate that is not intimately associated with carbonate from the samples. Next, the carbonate in the sample was rapidly dissolved by the addition of hydrochloric acid (HCl, 10 M) to liberate carbonate associated sulfate (CAS). Addition of HCl was stopped when no further evolution of carbon dioxide (bubbles) was observed with additional acid treatment. The supernatant was immediately filtered, and a saturated BaCl_2_ solution (~1.3 M BaCl_2_ in 0.05 M HCl) was added to induce the precipitation of BaSO_4_. After minimally 12 hours, the precipitated BaSO_4_ was recovered by centrifugation and decanting of the supernatant. The BaSO_4_ was washed with deionized water by three consecutive centrifugation-resuspension steps. For a discussion of the caveats of the CAS extraction methods, see [29]. After the CAS extraction, the sample was thoroughly washed and subsequently dried. Next a single-step Cr-reduction distillation was performed to obtain Cr-reducible sulfur [17,19]. Then the washing and drying step was repeated. Lastly, a STRIP distillation was applied to recover any residual sulfur fraction (RSF). The intriguing outcome of this sequential extraction (Table 5) was that the sulfur isotope composition of CAS and RFS-sulfur (average δ^34^S = +20.5 ‰) were almost identical and close to the composition of seawater sulfate, whereas CRS was strongly depleted in ^34^S (average δ^34^S = -47.6 ‰). We speculate that the residual sulfur pool, only accessible with the STRIP distillation, was marine barite. This would explain the similarity between the sulfur isotope composition of CAS, RSF, and seawater sulfate. From the methodological viewpoint, this result shows that STRIP-sulfur accessed a different sulfur pool than CRS, i.e. that no residual pyrite influenced the obtained results. Other research groups add the STRIP reagent directly to the Cr-reagent after the Cr-distillation is complete and obtain satisfactory results with this technique [18]. At this point, it should be noted that the STRIP reagent will extract all remaining sulfur from a sample in a sequential leaching procedure. Thus, if barite is one of the specifically targeted sulfur phases, a leaching step that is more selective would be indicated, for example the barite dissolution/re-precipitation technique using a chelating agent [30], which could then be followed by the final STRIP step.

**Table 5:** Sequential distillation of unconsolidated marine sediment

**Sample**	**δ**^**34**^**S**_**CAS**_**‰**	**δ**^**34**^**S**_**CRS**_**‰**	**δ**^**34**^**S**_**RSF**_**‰**
Eastern Equatorial Pacific sediments	Carbonate associated sulfate	Chromium reducible sulfur	Residual sulfur fraction extracted with STRIP distillation
Sediment A	20.9	-47.8	18.4
Sediment B	22.1	-45.8	19.5
Sediment C	21.3	-49.1	20.6

### Application to Aarhus Bay - a pilot study

*Motivation:* Exciting new hypotheses in sulfur biogeochemistry (e.g. [2]) highlight the fact that progress in our understanding of sulfur cycling without closed sulfur and sulfur isotope mass balances can hardly be achieved because pivotal sulfur pools and fluxes may have escaped detection when following standard analyses. Station M1 in Aarhus Bay is a popular sampling site with well characterized rates of both organoclastic sulfate reduction and sulfate reduction coupled to the anaerobic oxidation of methane. We chose this site to apply the STRIP distillation to pore-water samples and assess the sulfur mass balance of this system.

*Sampling and analyses:* An approximately 2 meter long gravity core was retrieved during a short sampling cruise in May 2011. The core was transported to laboratories at Aarhus University where pore-water was extracted on the same day. The gravity core was sub-sampled by cutting 20 cm whole-round sections, one end of the sub-section was capped and the other end was covered tightly with plastic wrap ensuring no air pockets between the plastic foil and sediment surface. Pore-water was extracted using Rhizon® pore-water samplers inserted through a small hole punctured in the plastic wrap (for description and supplier, see p 99–100 in [31]). Pore-water volumes extracted ranged from 10 ml from the deepest sample to ~50 ml in the shallowest samples. Once pore-water extraction was complete, the pore-water was split into sub-samples for i) total sulfur concentration and isotope composition, ii) sulfate concentration, iii) sulfide concentration, iv) thiosulfate concentrations and v) sulfate/sulfide/‘other’ S isotope composition (Table 6). Weights and volumes were noted at every step. Sulfate, sulfide, and thiosulfate concentrations were analyzed by standard methods (ion chromatography [31], diamine/spectrophotometric methods [32], and bimane/HPLC [33], respectively). Series v samples were treated sequentially to separate sulfide and sulfate from any remaining unidentified S-species in solution. First, ZnS was separated from the sample by vacuum filtration through a 0.2 μm filter. The ZnS was dried on the filter paper in a 50°C oven overnight, after which it was carefully removed from the filter and retained for S isotope analysis. With a modified vacuum filtration system, the filtrate was directly collected in clean 50 ml vials that were subsequently acidified with 0.1 ml ultraclean HCl, after which the sample was flushed with a 10:90 CO_2_:N_2_ gas mixture for one hour to ensure that no sulfide remains in the sample. Next, a sub-sample was transferred to a centrifuge tube and a saturated solution of BaCl_2_ (~1.3 M BaCl_2_ in 0.05 M HCl) was added to precipitate sulfate as BaSO_4_. The following day, the samples were centrifuged and the supernatant was pipetted off into an open syringe and filtered through a 0.2 μm syringe filter into a new centrifuge tube. The BaSO_4_ precipitate was washed several times, dried overnight in a 50°C oven and retained for sulfate-S isotope analysis. Series i and the final filtrate of the series v samples were dried and distilled using the STRIP distillation with a 5 ml AgNO_3_ trap.

**Table 6:** Aarhus Bay sampling scheme

	**Sample**	**Container type**	**Volume (ml)**	**Treatment**
i	Total S	Clear glass vial	1.5	0.25 ml 20% ZnAc
ii	Sulfate concentration	Cryovial	0.5	Flushed with CO_2_ to remove sulfide
iii	Sulfide concentration	Clear glass vial	1	0.20 ml 20% ZnAc
iv	Thiosulfate and sulfite	Brown glass vial	0.5	Bimane
v	Sulfate/sulfide/‘other’ S isotope composition	N_2_ flushed 50 ml glass crimp top vial	> 10 ml	2 ml 20% ZnAc

**Figure 3:**
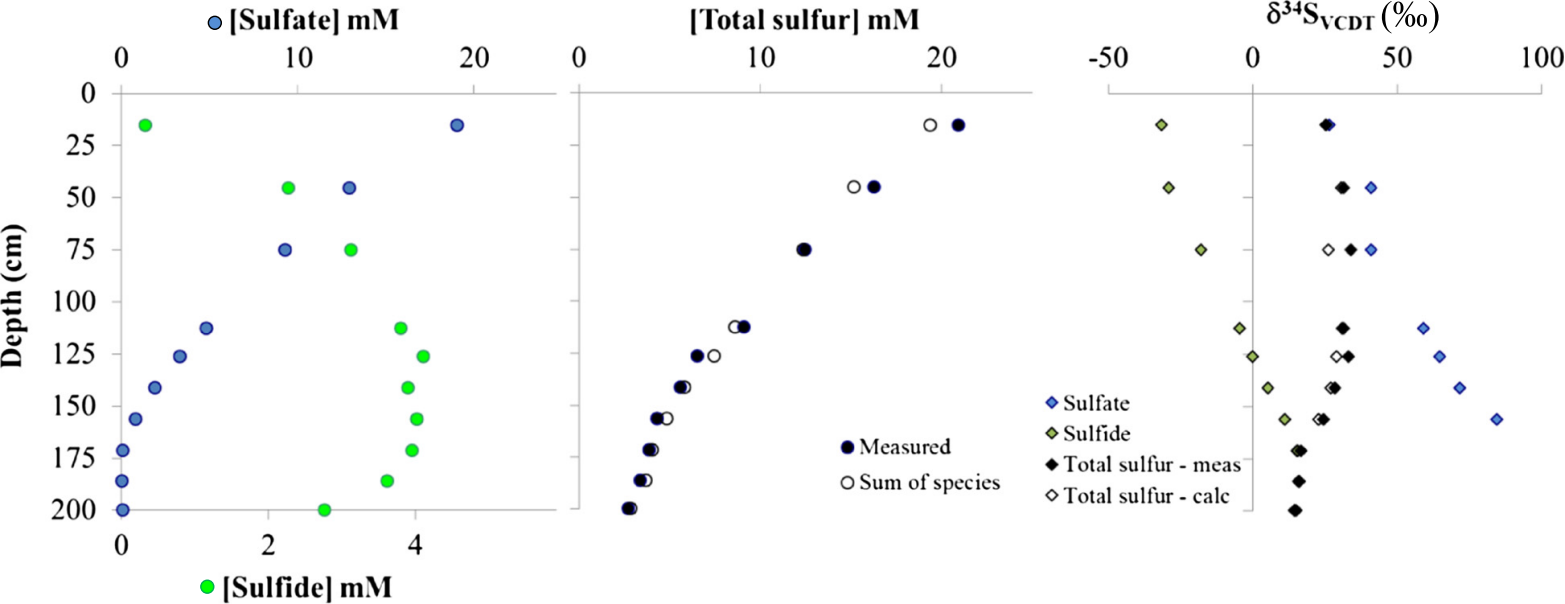
Sulfur species and sulfur isotope data from Aarhus Bay pore-water study.

**Figure 4:**
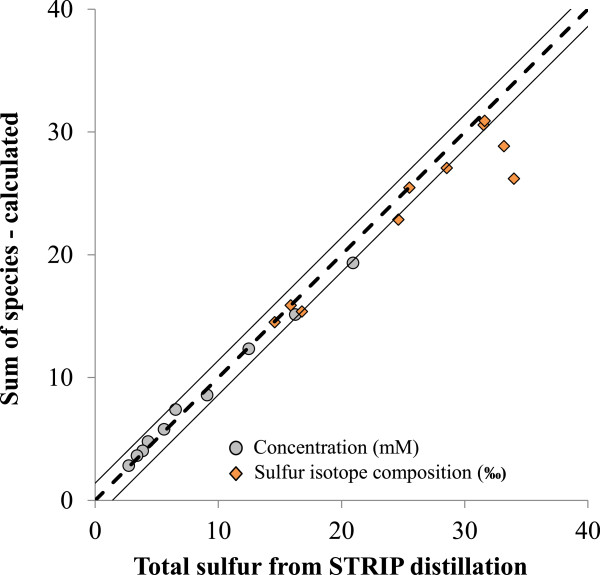
**Comparison of measured vs. calculated total sulfur concentrations and total sulfur isotope composition.** Dashed line represents a 1:1 relationship. Deviations from this line represents a discrepancy between the sulfur mass balance and the measured values. Error margin is ± 1.4 permil (2SD for 5 ml AgNO_3_ trap).

*Sulfur isotope mass balance of pore-water at station M1 in the Aarhus Bay:* Total sulfur concentrations, determined via the STRIP distillation method, decrease with increasing depth in the sediment. There is strong agreement between the total sulfur concentrations determined from the STRIP distillation with the sum of the individually measured sulfur pools (sulfate + sulfide + thiosulfate) with less than 10% discrepancy between the two values throughout the profile (Table 7, Figure 3). The sulfur isotope composition of the ‘total sulfur’ (δ^34^S_TSmeas_) samples also provide a good match with the total sulfur isotope composition calculated (δ^34^S_TScalc_) from the sulfate and sulfide fractions (Figure 4). Two total sulfur sulfur-isotope composition data points are obvious outliers when comparing δ^34^S_TSmeas_ vs. δ^34^S_TScalc_. The calculated total sulfur isotope composition of the sample from 75 cm shows the greatest deviation from the measured counterpart. This is likely the result of poor sample handling/labeling during the preparation of the sulfate-sulfur isotope sample. First, this sample has a nearly identical sulfate-sulfur composition as the sample from 45 cm (Figure 3). Second, because the volume and weight is recorded at every stage of sample preparation, we can gravimetrically back-calculate the pore-water sulfate concentration for any BaSO_4_ sample that was prepared. When this is done for the 75 cm sample a sulfate concentration of 14.4 mM is obtained, again nearly identical to that calculated for the 45 cm sample (14.5 mM calculated, 12.9 mM measured) and far removed from the measured sulfate concentration for the 75 cm sample ([SO_4_] = 9.2 mM, Table 7). Together, these observations strongly indicate that sulfate-sulfur isotope sample at 75 cm is corrupted, which comprises the δ^34^S_TScalc_ value and so we neglect this sample from further consideration.

**Table 7:** Summary of results from the Aarhus Bay pore-water study

**Sample ID**	**Depth (cm)**	**Total sulfur (mM)**	**Sulfate (mM)**	**Sulfide (mM)**	**Thiosulfate (mM)**	**δ**^**34**^**S**_**TSmeas **_**‰**	**δ**^**34**^**S**_**SO4 **_**‰**	**δ**^**34**^**S**_**HS-**_**‰**
1	15.0	20.9	19.0	0.3	0.011	25.5	26.5	-31.6
2	45.0	16.2	12.9	2.3	0.000	31.5	41.0	-29.1
3	75.0	12.5	9.2	3.1	0.011	34.0	41.1	-17.8
4	112.3	9.1	4.8	3.8	0.016	31.6	59.2	-4.6
5	126.0	6.5	3.3	4.1	0.008	33.2	64.7	0.1
6	141.0	5.6	1.9	3.9	0.014	28.5	71.8	5.4
7	156.0	4.3	0.8	4.0	0.012	24.6	84.7	11.2
8	171.0	3.8	0.08	4.0	0.011	16.8	-	15.7
9	185.8	3.4	0.02	3.6	0.018	15.9	-	16.1
10	199.5	2.7	0.07	2.8	0.023	14.6	-	15.0

-Samples below 150 cm did not produce enough BaSO_4_ for sulfur isotope analysis.

Looking at the remaining data, we are still left to consider an outlier at 126 cm and the general observation that although the rest of the data fall within two standard deviations of the expected 1:1 trend (Figure 4), half of the data lie at the very edge of the error margin. At first glance, this deviation might give the impression that an unidentified sulfur pool exists that contributes to the shift in δ^34^S. However, if we consider the total sulfur concentrations for these samples it becomes evident that the concentrations of the total sulfur samples are smaller than the sum of the sulfur species, the opposite of what one should observe if a ‘missing’ sulfur pool existed in this depth range (Table 8, Figure 3). In addition, there is the observation that the sulfur isotope compositions of total sulfur are heavier (Table 8; Figure 4) than the value of the sum of all species. With the caveat that we cannot predict the isotope fractionation processes for biochemical reactions between unknown sulfur pools and yet-undeciphered sulfur disproportionation pathways, we propose that typically, one would expect that sulfate is the isotopically heaviest pool, and that any other missing sulfur intermediates would likely have lighter values, resembling more the isotope composition of sulfide. In such a case the δ^34^S_TSmeas_ should be lighter than the δ^34^S_TScalc_, again the opposite of what is observed.

**Table 8:** Measured vs. calculated sulfur pools for the Aarhus Bay pore-water study

**Sample ID**	**Depth (cm)**	**Total sulfur (mM)**	**Sum of species (mM)**	**δ**^**34**^**S**_**TSmeas **_**‰**	**δ**^**34**^**S**_**TScalc **_**‰**
1	15.0	20.9	19.3	25.5	25.5
2	45.0	16.2	15.1	31.5	30.6
3	75.0	12.5	12.4	34.0	26.2
4	112.3	9.1	8.6	31.6	30.9
5	126.0	6.5	7.4	33.2	28.9
6	141.0	5.6	5.8	28.5	27.1
7	156.0	4.3	4.8	24.6	22.9
8	171.0	3.8	4.0	16.8	15.4
9	185.8	3.4	3.6	15.9	15.9
10	199.5	2.7	2.8	14.6	14.5

The use of a smaller (5 ml) AgNO_3_ trap in the STRIP distillation (Table 3), as was used in the Aarhus Bay study, tends to yield heavier δ^34^S_TSmeas_, and is likely the cause for the offset in the data. In addition, although each of the samples in question was distilled on a separate day, three were distilled on the same hot plate (on different days), suggesting that the poor data quality might also be due to a lower distillation temperature or loss of H_2_S from the assembly. This finding highlights the importance of good temperature control for the distillation assemblies and for the use of a larger, at least 10 ml, AgNO_3_ trap. The STRIP distillation of the final filtrate of the series v samples did not yield any sulfur, which in combination with the good match between the total sulfur and sum of all species profiles demonstrates that there is no major “missing sulfur pool” in the pore-water constituents at station M1 in the Aarhus Bay.

## Summary and conclusions

The STRIP distillation method remains a highly useful technique, with many applications. Although there are other options, like MC-ICPMS, for the sulfur isotope analysis of complex matrix samples of very low sulfur content, the STRIP distillation method provides a straightforward, easily accessible technique. In addition, the use of this method to convert ^35^S-sulfate tracer to ^35^S-sulfide tracer has immediate application in the elucidation of newly hypothesized and yet to be discovered sulfur intermediate pathways in microbial sulfur cycling. The potential exists to produce isotopically labeled elemental sulfur, as H_2_S is known to oxidize to elemental sulfur in the condensing column in the presence of nitrate [6], either by using the generated radiolabeled H_2_S or by using a modification of the STRIP distillation method to produce directly elemental sulfur. Stable isotope enriched elemental sulfur (^34^S-elemental S) is commercially available, but there is no such supply available for ^35^S-elemental sulfur. The STRIP distillation is also a powerful tool when applied towards total sulfur or in sequential combination with other extraction techniques where it can be used to complete the sulfur mass balance of a sample or system. Over the decades, many adjustments and variations of this method have been employed and the future utility of the STRIP distillation method, especially in the field of sulfur (isotope) biogeochemistry remains strong and promising.

## Competing interests

The authors declare that they have no competing interests.

## Authors’ contributions

GLA carried out most experiments and drafted the manuscript with input from all authors. GLA and BB designed the experiments and interpreted the results. IAM measured the sulfur intermediate species and assisted with Aarhus Bay study sample collection. HR managed the logistics for the Aarhus Bay study, assisted Aarhus Bay study sample collection and contributed the major sulfur species concentration measurements. All authors read and approved the final manuscript.
